# Chronic choriocapillaris ischemia in dilated vortex vein region in pachychoroid neovasculopathy

**DOI:** 10.1038/s41598-021-95904-9

**Published:** 2021-08-11

**Authors:** Hidetaka Matsumoto, Junki Hoshino, Ryo Mukai, Kosuke Nakamura, Shoji Kishi, Hideo Akiyama

**Affiliations:** Department of Ophthalmology, Gunma University Graduate School of Medicine, 3-39-15 Showa-machi, Maebashi, Gunma 371-8511 Japan

**Keywords:** Diseases, Eye diseases, Macular degeneration

## Abstract

We evaluated choroidal congestion using multimodal imaging in pachychoroid neovasculopathy (PNV). In a retrospective case series of 100 eyes of 99 treatment-naïve PNV patients, their clinical records were reviewed and the corresponding multimodal imaging studies were analyzed. We assessed areas of choriocapillaris filling delay which overlapped with dilated outer choroidal vessels, choroidal neovascularization (CNV), and retinal pigment epithelium (RPE) atrophy. The study subjects were 78 men (78.8%) and 21 women (21.2%). The mean patient age was 67.5 ± 10.5 years. On indocyanine green angiography, all eyes showed choriocapillaris filling delay in the early phase. Dilated outer choroidal vessels were demonstrated in all eyes by en face optical coherence tomography. The areas of choriocapillaris filling delay overlapped extensively with that of dilated outer choroidal vessels. All eyes showed CNV localized within the sites of choriocapillaris filling delay. RPE atrophy was noted in 71 eyes (71.0%), and 68 of these (95.8%) had RPE atrophy within the areas showing choriocapillaris filling delay. These findings indicate that chronic choriocapillaris ischemia secondary to vortex vein congestion may lead to CNV development as well as RPE atrophy in eyes with PNV.

## Introduction

Pachychoroid spectrum diseases were recently described as a new clinical entity comprised of macular disorders^1^. The term “pachychoroid” denotes an abnormal choroidal thickness increase, often with dilated choroidal vessels^1^. Central serous chorioretinopathy (CSC) typifies the pachychoroid disease spectrum^2^. Type 1 choroidal neovascularization (CNV) can reportedly develop in eyes with long-standing CSC and masquerade as neovascular age-related macular degeneration (AMD)^3,4^. Pang and Freund proposed the diagnosis of “pachychoroid neovasculopathy (PNV)” for eyes with Type 1 neovascularization associated with choroidal thickening in the absence of characteristic AMD or degenerative changes^5^. They found that PNV may eventually progress to polypoidal choroidal vasculopathy (PCV)^5^. These macular diseases, including CSC, PNV, and PCV, are widely regarded as being within the pachychoroid spectrum.

Choriocapillaris filling delay, choroidal vascular hyperpermeability (CVH), and dilated choroidal vessels are findings that have all been demonstrated in CSC^6–8^. Our recent investigations revealed asymmetric vortex veins (outer choroidal vessels) to be common in CSC, as well as markedly dilated dominant vortex veins, corresponding to sites of choriocapillaris filling delay in the early phase of indocyanine green angiography (ICGA)^9,10^. Furthermore, Pang et al., studying CSC, used ultra-widefield ICGA to demonstrate dominant vortex veins to be dilated from the distal end to the ampulla^11^. These findings indicate vortex vein congestion to be a major cause of CSC.

We used en face optical coherence tomography (OCT) imaging of the choroid to demonstrate that anastomosis between superior and inferior vortex veins is a common feature of pachychoroid spectrum diseases such as CSC, PNV, and PCV^12–14^. Anastomosis between superior and inferior vortex veins is reportedly secondary to vortex vein congestion^15,16^. Vortex vein congestion might thus be a common pathophysiology in pachychoroid spectrum diseases. Herein, we evaluated choroidal congestion as a possible cause of CNV in PNV employing multimodal imaging including ICGA.

## Results

**Figure 1:**
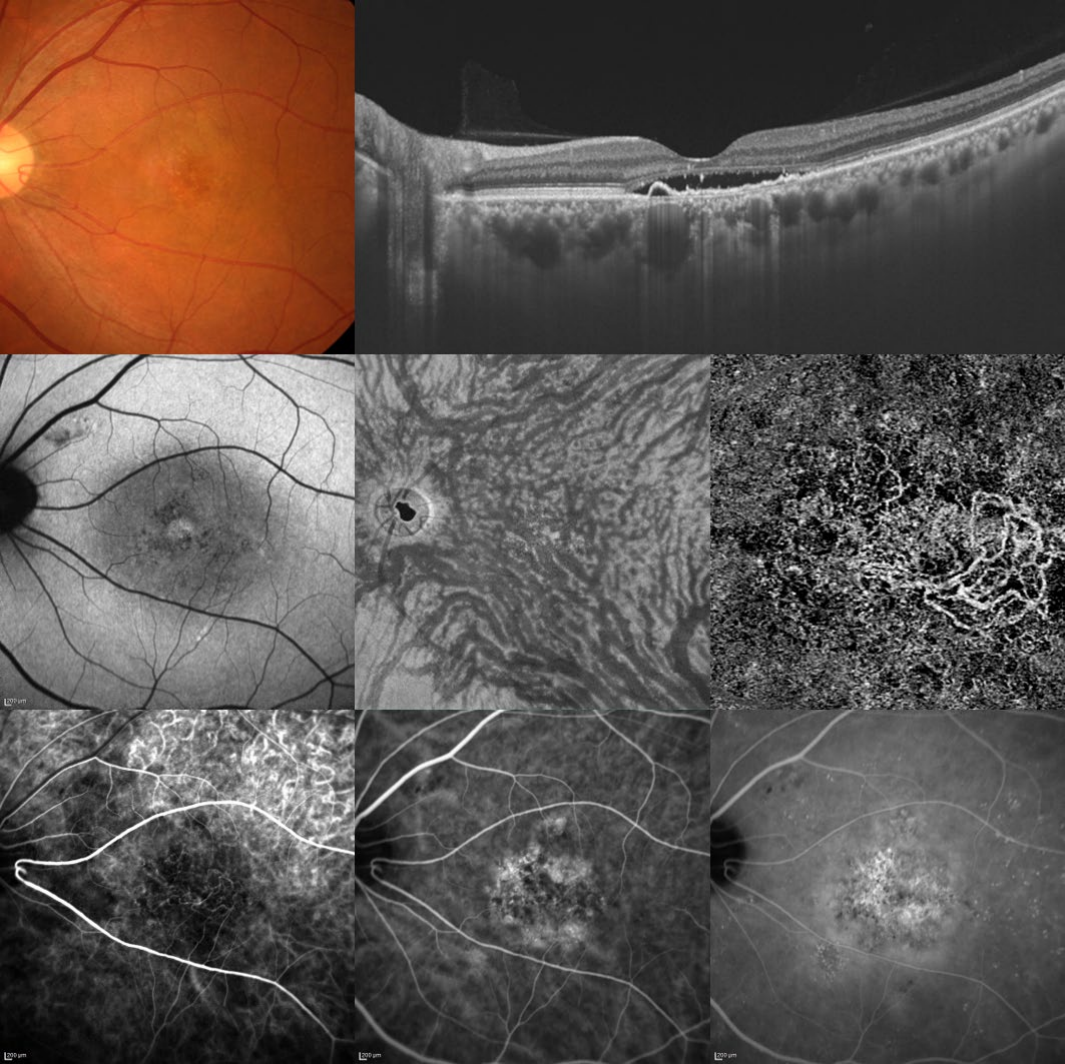
A 55-year-old man was diagnosed with pachychoroid neovasculopathy. Best-corrected visual acuity in the left eye was 0.30 logarithm of the minimum angle of resolution unit. Top left: The color fundus photograph shows retinal pigment epithelium (RPE) alteration in the macular and peripapillary areas. Top right: The horizontal optical coherence tomography (OCT) B-scan, through the fovea, shows dilated outer choroidal vessels (vortex veins) and shallow irregular RPE detachment accompanied by serous retinal detachment. The central choroidal thickness is 444 µm. Middle left: The fundus autofluorescence image shows hypoautofluorescent areas corresponding to RPE atrophy in the peripapillary area. Middle center: The en face OCT image (12 × 12 mm) shows dilated vortex veins and anastomoses between superior and inferior vortex veins. Middle right: The OCT angiography image (3 × 3 mm) shows a network of vessels comprising choroidal neovascularization (CNV) in the macular area. Bottom left: The early-phase indocyanine green angiography (ICGA) image shows geographic filling delay of the choriocapillaris and the network vessels of CNV in the macular area. The areas of choriocapillaris filling delay fully correspond to the dilated vortex vein region in the en face OCT image. CNV and RPE atrophy are localized within the areas of choriocapillaris filling delay. Bottom center: The ICGA image shows dilated vortex veins. The anastomoses between superior and inferior vortex veins are difficult to visualize. Bottom right: The late-phase ICGA image shows choroidal vascular hyperpermeability (CVH) and leakage from the CNV. The areas of CVH fully correspond to the dilated vortex vein region on the en face OCT image.

**Figure 2:**
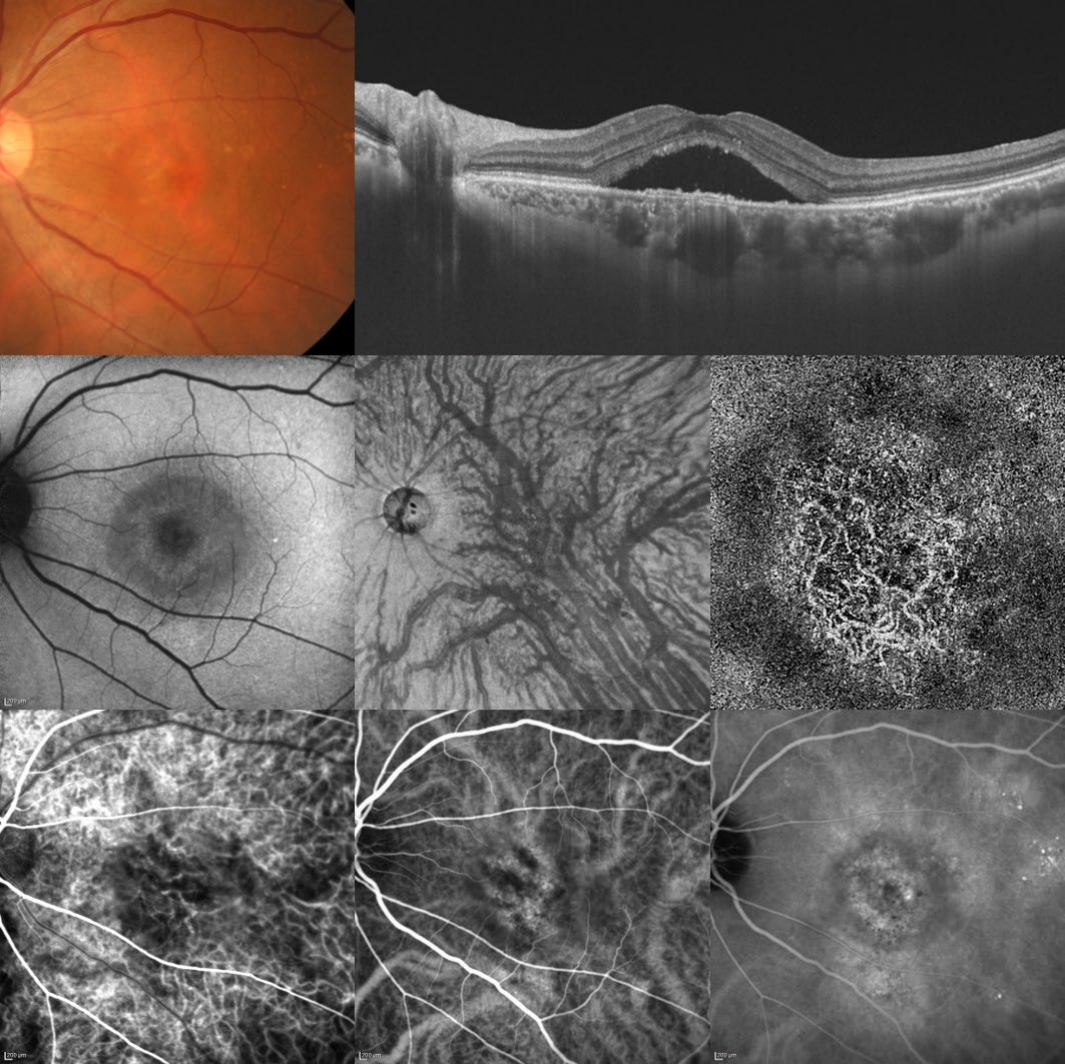
A 61-year-old woman was diagnosed with pachychoroid neovasculopathy. Best-corrected visual acuity in the left eye was -0.08 logarithm of the minimum angle of resolution unit. Top left: The color fundus photograph shows retinal pigment epithelium (RPE) alteration in the macular area. Top right: The horizontal optical coherence tomography (OCT) B-scan through the fovea shows dilated outer choroidal vessels (vortex veins) and shallow irregular RPE detachment accompanied by serous retinal detachment. The central choroidal thickness is 422 µm. Middle left: The fundus autofluorescence image shows no hypoautofluorescent area corresponding to RPE atrophy. Middle center: The en face OCT image (12 × 12 mm) shows dilated vortex veins and anastomoses between superior and inferior vortex veins. Middle right: The OCT angiography image (3 × 3 mm) shows network vessels of choroidal neovascularization (CNV) in the macular area. Bottom left: The early-phase indocyanine green angiography (ICGA) image shows geographic filling delay of the choriocapillaris and network vessels of CNV in the macular area. The areas of choriocapillaris filling delay fully correspond to the dilated vortex vein region in the en face OCT image. CNV is localized within the area of the choriocapillaris filling delay. Bottom center: The ICGA image shows dilated vortex veins and anastomoses between superior and inferior vortex veins. Bottom right: The late-phase ICGA image shows choroidal vascular hyperpermeability (CVH) and leakage from the CNV. The areas of CVH largely overlap with the dilated vortex vein region in the en face OCT image.

Table 1 lists the demographic and clinical characteristics of our PNV patients. Figures 1 and 2 show representative cases. The study subjects were all Japanese. There were 78 men (78.8%) and 21 women (21.2%). The mean patient age was 67.5 ± 10.5 years. On ICGA, all 100 eyes showed choriocapillaris filling delay in the early phase, and regional CVH was noted in the late phase in 63 (63.0%). Dilated outer choroidal vessels were evident in all eyes on the en face OCT images. The consistency of overlapping between the areas of choriocapillaris filling delay and the dilated vortex veins was grade 3, grade 2 and grade 1 in 58 (58.0%), 37 (37.0%), and 5 (5.0%) eyes, respectively. The consistency of overlapping between the CVH and the dilated vortex vein areas was grade 3, grade 2 and grade 1 in 45 (71.4%), 17 (27.0%), and 1 (1.6%) eye, respectively. All 100 eyes had CNV localized within choriocapillaris filling delay areas. Retinal pigment epithelium (RPE) atrophy was observed in 71 eyes (71.0%), and 68 of these eyes (95.8%) showed RPE atrophy within the areas of choriocapillaris filling delay. Mean central choroidal thickness (CCT) was 309 ± 97 µm. CCT was greater in eyes with than in those without CVH and the difference was statistically significant (326 ± 98 vs. 280 ± 88 µm, *P* < 0.05). Anastomosis was detected between the superior and inferior vortex veins in 67 eyes (67.0%) on ICGA images, but in 97 eyes (97.0%) on the en face OCT images (*P* < 0.01).

**Table 1:** Demographic and clinical characteristics of patients with pachychoroid neovasculopathy.

Number of patients	99	
Number of eyes	100	
Age (years)	67.5 ± 10.5	
Male	78 (78.8%)	
Choriocapillaris filling delay	100 (100%)	
Overlap of filling delay area and dilated vortex vein region (Grade: eyes)	Grade 3: 58 (58.0%) Grade 2: 37 (37.0%) Grade 1: 5 (5.0%) Grade 0: 0	
CVH	63 (63.0%)	
Overlap of CVH area and dilated vortex vein region (Grade: eyes)	Grade 3: 45 (71.4%) Grade 2: 17 (27.0%) Grade 1: 1 (1.6%) Grade 0: 0	
CNV within the filling delay area	100 (100%)	
RPE atrophy	71 (71.0%)	
RPE atrophy within the filling delay area	68 (95.8%)	
Central choroidal thickness (µm)	309 ± 97	
Central choroidal thickness in eyes with CVH (µm)	326 ± 98	*P* = 0.017
Central choroidal thickness in eyes without CVH (µm)	280 ± 88	
Vortex vein anastomosis in ICGA images	67 (67.0%)	*P* < 0.001
Vortex vein anastomosis in en face OCT images	97 (97.0%)	

*CVH* choroidal vascular hyperpermeability, *RPE* retinal pigment epithelium, *CNV* choroidal neovascularization, *ICGA* indocyanine green angiography, *OCT* optical coherence tomography.

Grade 3: filling delay or CVH area is entirely involved in the dilated vortex vein region.

Grade 2: 50% or more of filling delay or CVH area overlaps with the dilated vortex vein region.

Grade 1: less than 50% of filling delay or CVH area overlaps with the dilated vortex vein region.

Grade 0: no tendency for overlapping between filling delay or CVH area and dilated vortex vein region.

## Discussion

One hundred eyes with treatment-naïve PNV were retrospectively investigated using multimodal imaging. PNV eyes showed choriocapillaris filling delay on ICGA, and RPE atrophy and CNV were localized within the affected area. The choriocapillaris filling delay and CVH areas corresponded well to that of dilated outer choroidal vessels seen on the en face OCT. Eyes with CVH had significantly greater CCT than those without CVH. Anastomosis between superior and inferior vortex veins was a common finding in eyes with PNV.

CSC, a pachychoroid spectrum disease, is characterized by choriocapillaris filling delay, dilated choroidal vessels, and CVH on ICGA images^6–8^. A recent study by our group, employing en face OCT, revealed the areas of choriocapillaris filling delay on early-phase ICGA to overlap with those of dilated vortex veins in eyes with CSC^10^. Our present investigation of PNV demonstrated areas of the choriocapillaris filling delay and CVH to correspond to the area of dilated outer choroidal vessels. Therefore, choriocapillaris filling delay, CVH, and dilated outer choroidal vessels might be features common to all pachychoroid spectrum diseases. The vortex veins, divided into four quadrants based on horizontal and vertical watersheds, serve as choroidal drainage routes that pass through the sclera^17^. Thus, in pachychoroid spectrum diseases, vortex vein congestion might develop at the sclera, which in turn would lead to dilated choroidal vessels, choriocapillaris filling delay, and CVH.

Lee et al. described attenuation and thinning of the choriocapillaris and Sattler vessels overlying dilated outer choroidal vessels (pachyvessels) in their investigation of the B-mode OCT features of eyes with PCV^18^. The authors speculated that the mechanism underlying CNV development in PCV involves choriocapillaris attenuation, which in turn produces a relatively ischemic environment at the RPE-Bruch membrane complex level, triggering the expressions of angiogenic factors^18^. Their OCT findings in eyes with PCV are consistent with the choriocapillaris filling delay observed on ICGA in this investigation. In our current study of PNV, we observed CNV to be localized within choriocapillaris filling delay areas in all eyes. Chronic choriocapillaris ischemia is thus associated with choroidal congestion, possibly leading to the development of CNV in eyes with PNV. Indeed, previous reports demonstrated choriocapillaris filling defects on ICGA or reduced choriocapillaris flow on OCT angiography (OCTA) in pachychoroid spectrum diseases^19,20^.

Miyake and colleagues studied the phenotypic/genetic differences between neovascular AMD and PNV including PCV^21^. They described patients with PNV as being significantly younger than those diagnosed with neovascular AMD^21^. It was also noteworthy in their study that genetic susceptibility to AMD was significantly lower in PNV than in neovascular AMD^21^. Hata et al. examined differences in intraocular vascular endothelial growth factor concentrations between neovascular AMD and PNV including PCV and found these concentrations to be lower in the latter^22^. These reports suggest that the mechanisms underlying CNV development might differ between these two ocular disorders. Choroidal circulation impairments may have a greater impact on CNV development in PNV than in neovascular AMD.

Pachychoroid pigment epitheliopathy, another pachychoroid spectrum disease, shows choroidal thickening and associated RPE abnormalities but there is no history of subretinal fluid^23^. RPE abnormalities are common in other pachychoroid spectrum diseases such as CSC, PNV, and PCV. In this study, RPE atrophy was localized within the area of the choriocapillaris filling delay on ICGA in eyes with PNV. These results indicate RPE abnormalities in pachychoroid spectrum diseases to possibly be related to chronic choriocapillaris ischemia due to choroidal congestion.

Jirarattanasopa and colleagues investigated choroidal thickness in the macula of CSC eyes employing OCT and found choroidal thickness to be greater in areas with CVH than in unaffected areas^24^. Moreover, Koizumi and colleagues noted that patients with PCV associated with CVH more frequently showed a thickened choroid than those without CVH^25^. In our present study, CCT was significantly greater in eyes with than in eyes without CVH. These results suggest CVH to possibly reflect the degree of choroidal congestion leading to choroidal thickening in the pachychoroid spectrum diseases.

As in our previous studies, anastomosis between superior and inferior vortex veins was found to be common in pachychoroid spectrum diseases including PNV^12–14^. These results raise the possibility that choroidal congestion might lead to collateral vessel formation between superior and inferior vortex veins, capable of compensating for the choroidal congestion characteristic of pachychoroid spectrum diseases^12–14^. The anastomosis detection rate was significantly higher when en face OCT was employed than when ICGA was the imaging modality. On ICGA, the angioflow in the choriocapillaris and the vessels in Sattler’s layer might hamper visualization of the angioflow of anastomotic vessels in Haller’s layer. Recently, collaborating with Spaide et al., we proposed a new clinical entity designated “venous overload choroidopathy”, as a hypothetical framework for CSC and allied disorders, which shows abnormalities including intervortex venous anastomoses.

This study has limitations. It was retrospective in nature and had a single-center design. Examinations employing angiography and OCT focused on the posterior pole of the fundus, a site which shows only the posterior portion of the choroidal circulation. The areas of choriocapillaris filling delay, CVH, and dilated outer choroidal vessels were subjectively determined. CCT measurements were carried out manually. This study lacked normal or diseased control groups. Moreover, because this study was cross-sectional, it was not proven that dilated vortex veins and/or choriocapillaris ischemia were primary, or even secondary, changes in PNV. All 100 of our subjects were Japanese, and the results may thus not be generalizable to a larger PNV population, including Caucasians and other racial or ethnic groups.

In conclusion, we observed dilation of the outer choroidal vessels, corresponding to the choriocapillaris filling delay and CVH in PNV. CNV and RPE atrophy were localized within areas showing choriocapillaris filling delay. These results suggest chronic ischemia of the choriocapillaris secondary to vortex vein congestion, possibly leading to the development of CNV and RPE atrophy in eyes with PNV.

## Methods

This study was performed in compliance with the Declaration of Helsinki, after obtaining approval from the Gunma University Hospital institutional review board. Informed consent was obtained from all individual participants included in the study. Also, all of our participants provided informed consent for publication of identifying information/images. We retrospectively assessed 100 eyes of 99 patients with previously untreated PNV. All 100 participants were followed from April 2017 through October 2020 at our institution, Gunma University Hospital.

All patients with pachychoroid spectrum diseases received complete ophthalmological examinations, including slit-lamp biomicroscopy with a noncontact fundus lens (SuperField lens; Volk Optical Inc., Mentor, OH), color fundus photography (Canon CX-1; Canon, Tokyo, Japan), fundus autofluorescence (FAF), fluorescein angiography (FA) and ICGA with an angle of 30 degrees (Spectralis HRA + OCT; Heidelberg Engineering, Heidelberg, Germany), as well as swept-source OCT (DRI OCT-1 Triton; Topcon Corp, Tokyo, Japan, and PLEX Elite 9000; Carl Zeiss Meditec, Dublin, CA, USA). We obtained B-mode images of the horizontal and vertical line scans (12 mm) through the fovea employing the DRI OCT-1 Triton. Next, cube data were collected with a raster scan protocol of 1024 (horizontal) × 1024 (vertical) B-scans, i.e., with coverage of the 12 × 12 mm area centered on the fovea, employing the PLEX Elite 9000. We obtained en face images from the vitreous to the choroidoscleral border with coronal slices based on a 3-dimensional dataset included in the inner software. Next, we carried out OCTA volume scanning, i.e., 300 × 300 pixels in the 3 × 3 mm area demonstrated by the PLEX Elite 9000. This OCTA protocol was based on an optical microangiography algorithm.

Herein, we defined clinical and anatomical features of the pachychoroid as pathologically dilated outer choroidal vessels (pachyvessels) on B-mode and/or en face OCT images. CCT was not included among the pachychoroid phenotype criteria because CCT is impacted by both refractive errors and age^26^. Furthermore, eyes with normal CCT can exhibit extrafoveal choroidal thickening at sites affected by CNV^27^. A diagnosis of PNV was made if CNV associated with pachyvessels was seen on FA, ICGA, and/or OCTA images. CNV findings on OCTA were present in the slab from the outer retina to the choriocapillaris. OCTA has been reported to be useful for detecting CNV under shallow irregular pigment epithelial detachments in the pachychoroid spectrum diseases^28,29^. For the purposes of this study, PNV was taken to mean pachychoroid neovasculopathy without polypoidal lesions.

We retrospectively compared sites of choriocapillaris filling delay and CVH on early and late phase ICGA images, respectively, with the region showing dilated outer choroidal vessels (pachyvessels) on the en face OCT images. The consistency of overlapping between the choriocapillaris filling delay or CVH areas and dilated vortex vein regions was graded as in our previous publication^10^, as follows: Grade 3: Filling delay or CVH area is entirely involved in the dilated vortex vein region. Grade 2: 50% or more of the filling delay or CVH area overlaps with the dilated vortex vein region. Grade 1: Less than 50% of filling delay or CVH area overlaps with the dilated vortex vein region. Grade 0: no tendency for overlapping between filling delay or CVH area and dilated vortex vein region. We then evaluated whether or not the areas of choriocapillaris filling delay included the regions of CNV and/or retinal pigment epithelium (RPE) atrophy. For PNV eyes showing multiple sites of RPE atrophy, we assessed whether all of these lesions were included in the areas of choriocapillaris filling delay. We also determined whether anastomoses were present between the superior and inferior vortex veins, using ICGA and en face OCT images. Vortex vein anastomosis was considered to be present if anastomotic vessels showed connections with the superior and inferior vortex veins at the horizontal watershed zone. There was no narrowing of anastomotic vessels toward the watershed zone. To study the vortex veins using en face OCT, we assessed the images obtained at a depth of every 8 µm in the choroid. RPE atrophy was diagnosed based on the results obtained by both color fundus photography and FAF showing hypofluorescent areas outside the CNV region. CCT was also measured on B-scan images employing a computer-based caliper measurement tool in the OCT system. We defined CCT as the distance between Bruch’s membrane and the margin of the choroid and sclera under the fovea. The areas of choriocapillaris filling delay, CVH, and dilated vortex veins, as well as the presence of anastomoses between the superior and inferior vortex veins, were judged by two experienced retinal specialists (H. M and J. H).

The Mann–Whitney U test was employed to compare unpaired CCT values. The chi-squared independence test was applied to determine differences in the rate of detecting anastomosis between superior and inferior vortex veins. All data analyses were performed using Excel 2016 (Microsoft, Redmond, WA, USA) with the add-in software Statcel4^30^. A *P* < 0.05 was considered to indicate a statistically significant difference. Age and CCT values are presented as means ± standard deviation.
